# Supplementary material for: Identification of photoperiod-induced specific miRNAs in the adrenal glands of Sunite sheep (Ovis aries)
Source: Front Vet Sci. 2022 Jul 22;9:888207. doi: 10.3389/fvets.2022.888207 (PMC9354845; doi:10.3389/fvets.2022.888207)
Supplement: Supplementary file 1 [file Data_Sheet_1.ZIP › Supplementary file/miRDeep2 file.zip!/miRDeep2 file/Know_Structure/SP42A2/oar-mir-127.pdf]

miRBase precursor : oar-mir-127
Total read count : 158411
oar-miR-127 read count : 156233
remaining reads : 2178

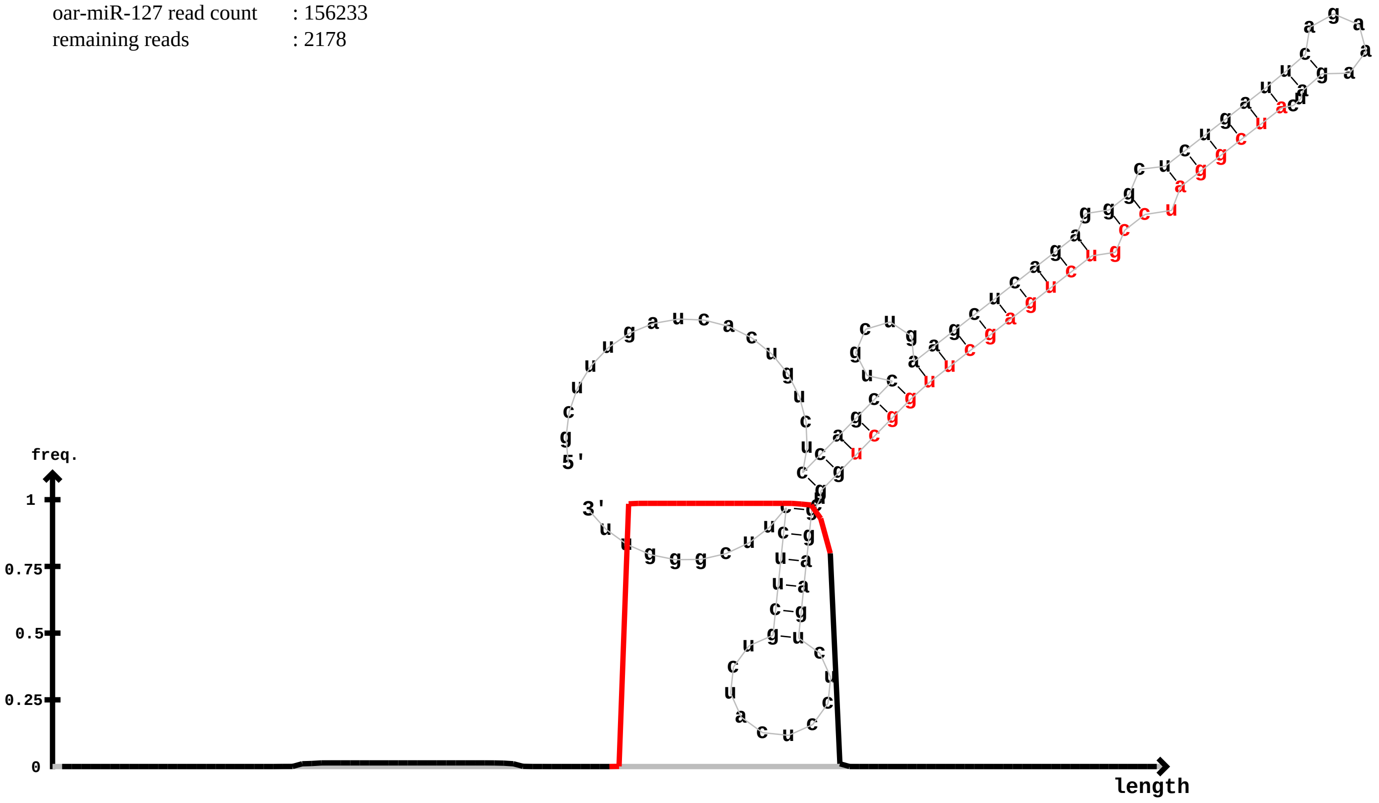

oar-miR-127

5'- gcuuugaucacugucuccagccugcugaagcucagagggcucugauucagaaagaucaucggauccgucugagcuuggcuggucggaagucuccucaucugcuuccucggguu -3'	exp		
................((((((.....(((((((((.((.((((((((.....))..)))))).)).)))))))))))))))..((((((.........))))))........	reads	mm	sample
.cuuugaucacugucuccagccug...	2	0	seq
...uugaucacugucuccagccug...	2	0	seq
....ugaucacugucuccagccug...	3	0	seq
......................ugcugaagcucagagggcucu..	2	0	seq
......................ugcugaagcucagagggcucug...	9	0	seq
......................ugcugaagcucagagggcucuga..	18	0	seq
......................ugcugaagcucagagggcucugau...	9	0	seq
......................ugcugaagcucagagggcucugauu..	5	0	seq
.......................gcugaagcucagagggcucug...	1	0	seq
.......................gcugaagcucagagggcucuga..	5	0	seq
.......................gcugaagcucagagggcucugau...	1	0	seq
.......................gcugaagcucagagggcucugauu..	4	0	seq
........................cugaagcucagagggcuc...	1	0	seq
........................cugaagcucagagggcuU...	1	1	seq
........................cugaagcucagagggcucu..	2	0	seq
........................cugaagcucagagggcucC..	1	1	seq
........................cugaagcucagagggcucug...	16	0	seq
........................cugaagcucagagggcucuga..	90	0	seq
........................cugaagcucagagggcucugau...	365	0	seq
........................cugaagcucagagggcucugauu..	1091	0	seq
........................cugaagcucagagggcucugauuc...	16	0	seq
........................cugaagcucagagggcucugauucaga..	1	0	seq
.........................ugaagcucagagggcucug...	2	0	seq
.........................ugaagcucagagggcucuga..	4	0	seq
.........................ugaagcucagagggcucugau...	9	0	seq
.........................ugaagcucagagggcucugauu..	143	0	seq
.........................ugaagcucagagggcucugauuc...	4	0	seq
..........................gaagcucagagggcucugau...	5	0	seq
..........................gaagcucagagggcucugauu..	250	0	seq
..........................gaagcucagagggcucugauuc...	88	0	seq
..........................gaagcucagagggcucugauuca..	8	0	seq
..........................gaagcucagagggcucugauucaga..	3	0	seq
...........................aagcucagagggcucugauu..	10	0	seq
...........................aagcucagagggcucugauuc...	1	0	seq
...........................aagcucagagggcucugauuca..	2	0	seq

```
gcuuugaucacugucuccagccugcugaagcucagagggcucugauucagaaagaucaucggauccgucugagcuuggcuggucggaagucuccucaucugcuuccuucggguu
...........................aagcucagagggcucugauucag................................................................    3       0       seq
...........................aagcucagagggcucugauucaga...............................................................    1       0       seq
.......................................................ucaucggauccgucugagcuugg....................................    1       0       seq
.......................................................ucaucggauccgucugagcuuggc...................................    1       0       seq
........................................................caucggauccgucugagcuug.....................................    1       0       seq
........................................................caucggauccgucugagcuugg....................................    1       0       seq
........................................................caucggauccgucugagcuuggc...................................    1       0       seq
.........................................................aucggauccgucugagcuugg....................................    26      0       seq
.........................................................aucggauccgucugagcuuggc...................................    51      0       seq
.........................................................aucggauccgucugagcuuggcu..................................    7       0       seq
..........................................................ucggauccgucugagcu.......................................    9       0       seq
..........................................................ucggauccgucugagcuu......................................    425     0       seq
..........................................................ucggauccgucugagcuug.....................................    537     0       seq
..........................................................ucggauccgucugagcuugg....................................    7943    0       seq
..........................................................ucggauccgucugagcuuggc...................................    20679   0       seq
..........................................................ucggauccgucugagcuuggcu..................................    124662  0       seq
..........................................................ucggauccgucugagcuuggcug.................................    1600    0       seq
..........................................................ucggauccgucugagcuuggcugg................................    14      0       seq
...........................................................cggauccgucugagcuugg....................................    9       0       seq
...........................................................cggauccgucugagcuuggc...................................    36      0       seq
...........................................................cggauccgucugagcuuggcu..................................    191     0       seq
...........................................................cggauccgucugagcuuggcug.................................    23      0       seq
...........................................................cggauccgucugagcuuggcugg................................    1       0       seq
............................................................ggauccgucugagcuugg....................................    1       0       seq
............................................................ggauccgucugagcuuggcu..................................    9       0       seq
............................................................ggauccgucugagcuuggcugg................................    2       0       seq
............................................................ggauccgucugagcuuggcuggu...............................    3       0       seq
```
